# Molecular profiles of blood from numerous species that differ in sensitivity to acute inflammation

**DOI:** 10.1186/s10020-024-01052-x

**Published:** 2024-12-28

**Authors:** David J. Gregory, Feifei Han, Peng Li, Marina A. Gritsenko, Jennifer Kyle, Frank E. Riley, Deborah Chavez, Vania Yotova, Renata H. M. Sindeaux, Mohamed B. F. Hawash, Fengyun Xu, Li-Yuan Hung, Douglas L. Hayden, Ronald G. Tompkins, Robert E. Lanford, Lester Kobzik, Judith Hellman, Jon M. Jacobs, Luis B. Barreiro, Wenzhong Xiao, H. Shaw Warren

**Affiliations:** 1 Department of Pediatrics, Massachusetts General Hospital, Boston, MA USA; 2 Harvard Medical School, Boston, MA USA; 3 Department of Surgery, Massachusetts General Hospital and Harvard Medical School, Boston, MA USA; 4 Biological Sciences Division, Pacific Northwest National Laboratory, Richland, WA USA; 5 Southwest National Primate Research Center, Texas Biomedical Research Institute, San Antonio, TX USA; 6 Centre Hospitalier Universitaire Sainte-Justine, Montréal, QC Canada; 7 Department of Biochemistry, University of Montréal, Montréal, QC Canada; 8 Department of Anesthesia and Perioperative Care, University of California, San Francisco, San Francisco, CA USA; 9 Program in Molecular and Integrative Physiological Sciences, Harvard T.H. Chan School of Public Health, Boston, MA USA; 10 Section of Genetic Medicine, Department of Medicine, University of Chicago, Chicago, IL USA; 11 Department of Human Genetics, University of Chicago, Chicago, IL USA; 12 Committee On Genetics, Genomics, and Systems Biology, University of Chicago, Chicago, IL USA; 13 Committee On Immunology, University of Chicago, Chicago, IL USA; 14 Department of Medicine, Massachusetts General Hospital, Boston, MA USA; 15 Present Address: Hospital for Sick Children, Toronto, Canada

**Keywords:** Inflammation, Animal models, Genomic response

## Abstract

Vertebrates differ over 100,000-fold in responses to pro-inflammatory agonists such as bacterial lipopolysaccharide (LPS), complicating use of animal models to study human sepsis or inflammatory disorders. We compared transcriptomes of resting and LPS-exposed blood from six LPS-sensitive species (rabbit, pig, sheep, cow, chimpanzee, human) and four LPS-resilient species (mice, rats, baboon, rhesus), as well as plasma proteomes and lipidomes. Unexpectedly, at baseline, sensitive species already had enhanced expression of LPS-responsive genes relative to resilient species. After LPS stimulation, maximally different genes in resilient species included genes that detoxify LPS, diminish bacterial growth, discriminate sepsis from SIRS, and play roles in autophagy and apoptosis. The findings reveal the molecular landscape of species differences in inflammation. This may inform better selection of species for pre-clinical models and could lead to new therapeutic strategies that mimic mechanisms in inflammation-resilient species to limit inflammation without causing immunosuppression.

**Supplementary Information:**

The online version contains supplementary material available at 10.1186/s10020-024-01052-x.

## Background

**Fig.1:**
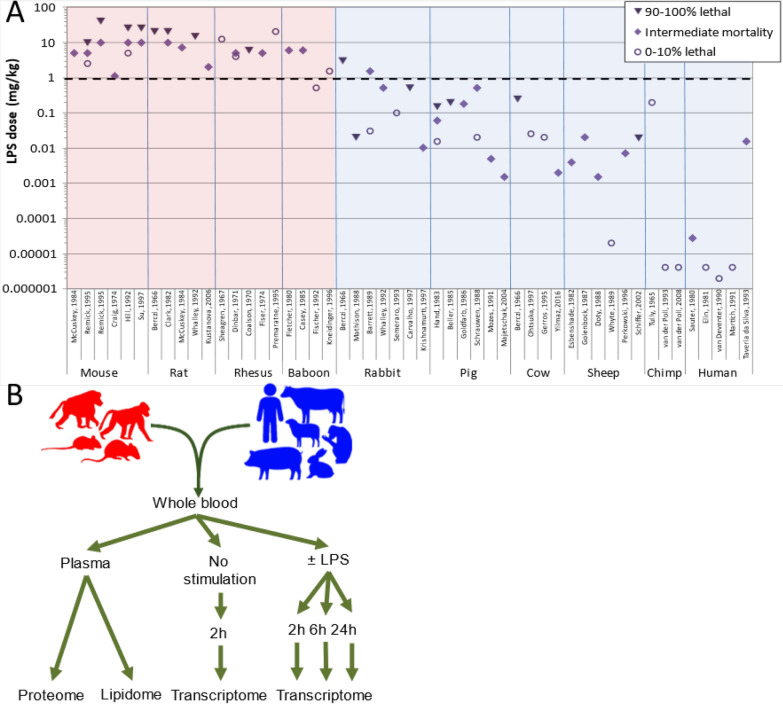
Rationale of study. **A** Lethal doses of LPS in different species. Values are selected from representative studies (shown on the x-axis) where LPS was injected i.p. or i.v. and survival reported. Wherever possible, studies using a range of doses of *E. coli* LPS injected as a single bolus are reported. Resilient species, defined here as those that survive a dose of 1 mg/kg body weight (dashed line), are indicated with red background; sensitive species are indicated with a blue background. References are given in Table S2. **B** Outline of study. Whole blood from 4–5 individuals of 4 resilient species (mouse, rat, rhesus, and baboon, indicated in red) and 6 sensitive species (rabbit, pig, cow, sheep, chimp, human, indicated in blue) was incubated ex vivo with 0, 10, 100, or 1000 ng/mL *E. coli* LPS for 2, 6, or 24 h followed by leukocyte mRNA. sequencing. MS/MS analysis of lipids and proteins was performed on plasma without ex vivo stimulation. In each case, data from blood of resilient species was compared to that from sensitive species

For decades, bacterial lipopolysaccharide (LPS, endotoxin) has been injected into different species to induce and study inflammatory responses that are intended to mimic human inflammation. Although there are substantial differences in preparations and administration**,** the bioactive moiety of LPS (lipid A) is generally conserved, so that it is possible to rank the sensitivity of species to LPS (Fig. 1A). These data indicate that species differ over a hundred thousand-fold in sensitivity to LPS. Rodents and some non-human primates used in many disease models are very resilient to LPS and require over a milligram LPS/kg to induce pathology. In contrast, humans fall on the end of extreme sensitivity. Most studies in humans have utilized a standardized reference endotoxin prepared by the FDA and administered to volunteers at a dose of 2–4 ng LPS/kg, which causes predictable flu-like symptoms, fever and cytokine release (Lowry 2005) Two documented cases of endotoxin injected into humans at higher doses (27 ng/kg and 17 µg/kg), caused shock and required intensive care support (Sauter and Wolfensberger 1980; Taveira da Silva et al. 1993).

While this extreme sensitivity of humans to inflammation may be an advantage to protect against live infectious challenges, it likely comes at a higher cost of secondary tissue damage (Read et al. 2008; Medzhitov et al. 2012; Råberg et al. 2007; Ayres et al. 2008; Jamieson et al. 2013) than for species that are more resilient. Ironically, a large segment of our pharmacopoeia consists of drugs designed to block innate inflammation, all which have the side effect of increased risk of infection. The mechanisms that some species utilize to minimize agonist-induced innate inflammation while apparently at the same time avoiding immunosuppression are poorly understood.

To better understand how different species respond to the same agonist challenge we compared the proteomics, HDL proteomics, and lipidomics of plasma and the transcriptomics of leukocytes in the whole blood of 10 species that vary widely in sensitivity to in vivo LPS challenge. We used carefully controlled and standardized protocols to collect the plasma and the whole blood of 5 mice, rats, rhesus monkeys, baboons, rabbits, pigs, cows, sheep, chimpanzees, and humans into heparinized tubes containing no or varied doses of LPS. Plasma and leukocytes were harvested at 2, 6, and 24 h and analyzed for protein or lipid and mRNA (Fig. 1B). For the purpose of analysis, we divided species into two groups: those “resilient” to LPS (in vivo sensitivity of > 1 mg/kg: mouse, rat, rhesus, baboon) and those “sensitive” to LPS (rabbits, pigs, cows, sheep, chimpanzees, and humans) (Fig. 1A). The terminology for underlying resilience to LPS challenge between species is not precise (Read et al. 2008). Baseline resilience to LPS differs from the resilient state induced by prior LPS challenge as well as from the ability to limit the damage caused by a given live microbial burden, both of which have been previously defined as tolerance (Read et al. 2008; Medzhitov et al. 2012; Råberg et al. 2007; Ayres et al. 2008; Jamieson et al. 2013; Cavaillon and Adib-Conquy 2006). We use “sensitivity” and “resilience” as the terminology for this article (Warren et al. 2010).

## Materials and methods

Animal details are summarized in Table S1.

Non-human primates: Blood samples from chimpanzees, baboons and rhesus macaques were obtained from the colony at the Southwest National Primate Research Center (SNPRC) at Texas Biomedical Research Institute. The animals were cared for in accordance with the Guide for the Care and Use of Laboratory Animals. SNPRC is accredited by AAALAC International. All protocols were approved by the institutional IACUC (protocols 1508 PC CJ MM SB and 525 PT). Animals were selected to be of approximately the same age and were naïve with regard to experimental procedures. Rhesus macaques were sourced from the SNPRC specific pathogen free colony and were negative for Herpes B Virus, Simian immunodeficiency virus, Simian Betaretrovirus (SRV), Simian T-Cell Lymphotrophic Virus. Animals were group housed in outdoor housing except during recovery from procedures, and animals were sedated prior to obtaining blood samples. Chimpanzee samples were obtained prior to the ban on chimpanzee research. Male animals were used because of the high demand of females as breeders, and because the total number of animals involved in the study was too small to demonstrate a significant difference between sexes.

Humans: Blood was obtained from five male human donors in heparin tubes. The protocol was approved by the University of Texas Health Science Center IRB (IRB# HSC20170139H).

Sheep, Pigs, Cows: Surplus blood, collected during routine veterinary check-ups, was used. 10 mL of blood were collected in heparin tubes from each of five adult female cows, sheep, and castrated pigs.

Mice: Because of the small volume of blood in a single mouse, each individual sample was a composite of blood pooled from 25 × 10 week old, male C57/BL6J male mice (Jackson Laboratories). Each sample was prepared on a different day. Blood was drawn by cardiac puncture under deep ketamine-xylazine in accordance with Massachusetts General Hospital IACUC protocol (2003N000329).

Rabbits: A total of five 22-week old male New Zealand White rabbits (Western Oregon Rabbit Co, Philomath, OR) were deeply anesthetized with inhaled isoflurane. Then, using sterile technique, the maximum attainable volume of blood was collected by cardiac puncture in accordance with University of California San Francisco IACUC protocol AN152939. Each individual sample was the blood from one rabbit.

Rats: A total of twenty 14-week old male Sprague–Dawley rats (Simonsen Laboratories, Gilroy, CA) were deeply anesthetized with inhaled isoflurane. Using sterile technique they underwent a small thoracotomy incision to expose the heart. Then, using sterile technique, the maximum attainable volume of blood was collected by cardiac puncture in accordance with University of California San Francisco IACUC protocol AN152955. Each individual sample was a composite of blood pooled from 4 rats.

### Ex vivo* stimulation*

In all cases, blood was collected into heparin tubes using meticulous pyrogen-free conditions. TruCulture tubes (Myriad RBM) containing either cell culture media only (“control”) or cell culture media plus 10 ng/mL, 100 ng/mL, or 1 μg/mL *E. coli* 0113 US national reference strain LPS (Rudbach et al. 1976) (gift of Anthony Rudbach) were prepared in advance, stored at − 20 °C or below, and thawed at room temperature immediately prior to use. 1 mL blood was added to each tube and samples were incubated for 2, 6 and 24 h at 37 °C with 5% CO_2_. After incubation, blood in TruCulture tubes was separated according to the manufacturer’s instructions with additional centrifugation at 800 × g for 10 min. Supernatants were saved and frozen at − 80 °C for cytokine analysis. The cell pellets were lysed using red blood cell lysis solution for 10 min followed by centrifugation at 2000 × g for 3 min, and remaining white blood cells were lysed in Qiazol and frozen at − 80 °C until RNA isolation. An additional aliquot of blood was immediately separated by centrifugation, without incubation in TruCulture tubes, and plasma stored at − 80 °C for proteomic analysis.

### Cytokine analysis

Plasma from mice and rats was assessed for the cytokines reported by Luminex technology (MAGPIX, Millipore Sigma), using commercially available beads (mice MCYTOMAG-70 K-13, rats RECYTMAG-65 K-12, non-human primates PRCYTOMAG-40 K-13, humans HCYTOMAG-60 K-13, Millipore-Sigma).

### RNA sequencing

RNA isolated from white blood cells (Qiazol, Qiagen) was quality controlled using A260/A280 ratio, 28S/18S rRNA ratio, and an RNA integrity summary score (Agilent RIN). Total RNA samples (250ngs, RIN >  = 7.0) were enriched for mRNA, fragmented, and converted into indexed cDNA libraries according to Illumina TruSeq Stranded mRNA protocol and quantity controlled using Agilent TapeStation. Samples were sequenced to at least 25 million of 2 × 50 bp paired-end reads (i.e. at least 25 million × 2 × 50 bp = 2.5 billion base pairs of data) and assessed using in-house QA/QC metric (Q2Solutions Expression Analysis). RNAseq data related to this study has been deposited in GEO, Accession GSE249010.

### Gene expression analysis

Sequencing quality was assessed additionally using FASTQC (v0.11.9). Genome assembly and transcriptome annotations of each species were downloaded from Ensembl (https://www.ensembl.org, v90). For each sample, firstly, reads were aligned to the genome assembly of the corresponding species using STAR (Patro et al. 2014) in the established bulk-RNA sequencing workflow of Bcbio (v1.2.1) (Chapman et al. 2020). Secondly, the abundance of the annotated genes of the species was estimated from the RNA-seq reads as Transcripts per Million (TPM) using Sailfish (Patro et al. 2014; Robert and Watson 2015). Thirdly, to compare the gene expression between species, the abundance of the genes in each species was then translated to that of the corresponding human orthologues. Here, only genes in a non-human species that had known orthologous human genes (Ensembl v90) were considered in further analysis. When m genes in a non-human species had n orthologous genes in human (m >  = 0, n >  = 1), the abundance of each of the human orthologues is assumed to be the sum of the abundance of the m genes divided by n. Names and abbreviations of the human orthologs are used to refer to each gene.

### Statistical analysis of the transcriptome data

The Limma package in R (Ritchie et al. 2015) was utilized to identify transcriptome features significantly different between sensitive and resilient species. RNA sequencing data were analyzed after quantile normalization. For the comparison of baseline expression levels between the two groups of sensitive and resilient species, a mixed effects model was used. Briefly, a linear model was used to fit the data, where the sensitive or resistant groups were fixed effects, and individuals of each species were considered as random effects, using the blocking factor/random effect function in Limma. An empirical Bayes function was then applied to leverage information across genes to improve the accuracy of variance estimation. Significant genes were identified with a cutoff of fold difference >  = 2 and a false discovery rate (FDR) <  = 0.05. To evaluate the expression changes in response to LPS stimulation in each species, the expression data for each dose of LPS stimulation (10, 100, and 1000 ng/mL LPS) were compared with those of the control condition (no LPS) separately at each of the time points (2, 6, and 24 h) using a paired analysis, and a cutoff of fold change >  = 2 and an FDR <  = 0.05 was used. For the comparison of the differences in gene response to LPS stimulation between the groups of sensitive and resilient species at each time point, the differences in fold changes between the stimulation (10 ng/mL LPS) and the control were first calculated, followed by analysis using a likewise mixed effects model; significant genes between the two groups were identified with a cutoff of the difference in the fold changes >  = 1.5 and an FDR <  = 0.05. In comparisons of all resilient species vs all sensitive species, a single “missing” annotation was tolerated in the more strongly expressed or induced class, to accommodate incompleteness in genome annotations. Ingenuity Pathway Analysis (Calvano et al. 2005) was used to identify changes in activity of upstream regulators, which may help explain the observed difference in gene expression between sensitive and resilient species. The uncorrected, Fisher’s Exact Test P-value for the overlap and Activation Z-score were calculated as previously described (Krämer et al. 2014).

### Global plasma proteome analysis

Plasma samples for proteome analysis were collected simultaneously with leukocytes for transcriptome analysis. 30 µL plasma from each animal was pooled (150 µL total) and processed through reduction and tryptic digestion to the peptide form as previously described (Jarsberg et al. 2021). To avoid introducing bias, no affinity depletion was utilized in this study. Instrument analysis was performed using a platform based upon liquid chromatography mass spectrometry (LC–MS) to globally identify and quantify plasma peptide and their corresponding proteins within each sample and species as previously described (Deatherage Kaiser et al. 2020). Data generated was based upon label free peak intensity utilizing the MaxQuant data analysis pipeline as previously described (Alcazar et al. 2020) providing protein level quantitative data (LFQ values) for subsequent comparisons. Data from each unique species LC–MS analysis was searched independently from their respective RefSeq species and subsequently mapped onto a universal human protein for direct species comparison as previously denoted.

### Statistical analysis of the proteome data

Determination of discriminating proteins between sensitive and resilient species was based upon statistical comparisons utilizing the generated protein level quantitative values (LFQ) and utilized the DAnTE InfernoRDN analysis tool (Polpitiya et al. 2008). Specifically, each comparison was comprised of three mutually exclusive approaches. Protein abundances were first subjected to ANOVA analysis for generation of a p value, from which a < 0.05 threshold was utilized for an initial capture of discriminating proteins of interest. Secondly, proteins which did not have sufficient replicate abundances to generate a p value were compared via a fold-change abundance threshold (± 3.0 in log2 phase, minimum of 4 occurrences) resulting in capture of discriminating proteins of interest. Finally, proteins for which we were unable to generate a fold-change value, i.e. those which were present/absent in either the sensitive or resilient group, were captured and termed discriminating if they were detected in a minimum of 3 species.

### HDL proteome analysis

Isolation of HDL proteins from plasma for LC–MS analysis followed previously published protocols (Henderson et al. 2016). Initial starting amount was ~ 300 µl of plasma in biological triplicate. Following density gradient and ultracentrifugation steps, and removal of the HDL containing layer, desalting using Dialysis/Amicon 3 K ultracentrifugal filters was used to remove KBr, and Bradford Assay was used to determine protein concentration. Remaining protein processing to peptide form and LC–MS analysis and data analysis was performed identical to global as described above.

### Plasma lipidome analysis

For plasma, 50 μL was used for lipid extraction using a modified Folch extraction (Folch et al. 1957), the MPLEx protocol (Nakayasu et al. 2016). Details of the sample processing were as previously described (Kyle et al. 2021) to obtain a total lipid extract (TLE) for analysis. TLEs were analyzed as outlined in Kyle et al*.* (Kyle et al. 2017) and as previously described (Kyle et al. 2021) utilizing a Waters Acquity UPLC H class system interfaced with a Velos-ETD Orbitrap mass spectrometer for LC–ESI–MS/MS analyses. LC–MS/MS lipidomics data were analyzed using LIQUID (Lipid Informed Quantitation and Identification) (Kyle et al. 2017) and as previously described (Kyle et al. 2021). To facilitate the quantification of lipids, lipids identified from the MS/MS data from each analysis were then aligned based on their identification, *m/z,* and retention time using MZmine 2 (Pluskal et al. 2010). Aligned features were manually verified and peak apex intensity values were exported for subsequent statistical analysis.

### Targeted proteome analysis:

Selective Reaction Monitoring (SRM) was performed on the panel of primate plasma samples as described above. Crude heavy peptides labeled with 13C/15N on C-terminal lysine and arginine were purchased from New England peptides (Gardner, MA). Trypsin digested samples that had been stored at − 80 °C until use were processed as previously described (Shi et al. 2013). For each sample the digested peptides were diluted to 0.2 µg/µL containing standards at a final concentration of 250 fmol/µL for 11 protein standards and 500 fmol/µL for 12 proteins. All the samples were analyzed with a nanoACQUITY UPLC® system (Waters Cooperation, Milford, MA) coupled online to a TSQ Vantage triple quadrupole mass spectrometer (Thermo Scientific, San Jose, CA). The LC-SRM platform was configured and utilized as previously described (Nielson et al. 2016). Peptides used were DDKPTLQLESVDPK (IL-1β), VNLLSAIK (TNF), ESLLEDFK (IL-10), FLELAYR (G-CSF).

SRM data acquired on the TSQ Vantage were analyzed using Skyline software (MacLean et al. 2010). Peak detection and integration were determined based on retention time and the relative SRM peak intensity ratios across multiple transitions between light peptide and heavy peptide standards (He et al. 2014). All the data were manually inspected to ensure correct peak assignment and peak boundaries. The peak area ratios of endogenous light peptides and their heavy isotope-labeled internal standards (i.e., L/H peak area ratios) were then automatically calculated by Skyline, and the average peak area ratios from all the transitions were used for quantitative analysis of the samples. For targets that had more than one surrogate peptide, correlation graphs were plotted to verify a strong correlation and ultimately the peptide that had the most sensitive response was selected for obtaining quantitative values.

## Results

### Comprehensive profiling of plasma proteomics, HDL proteomics, and lipidomics

**Fig. 2:**
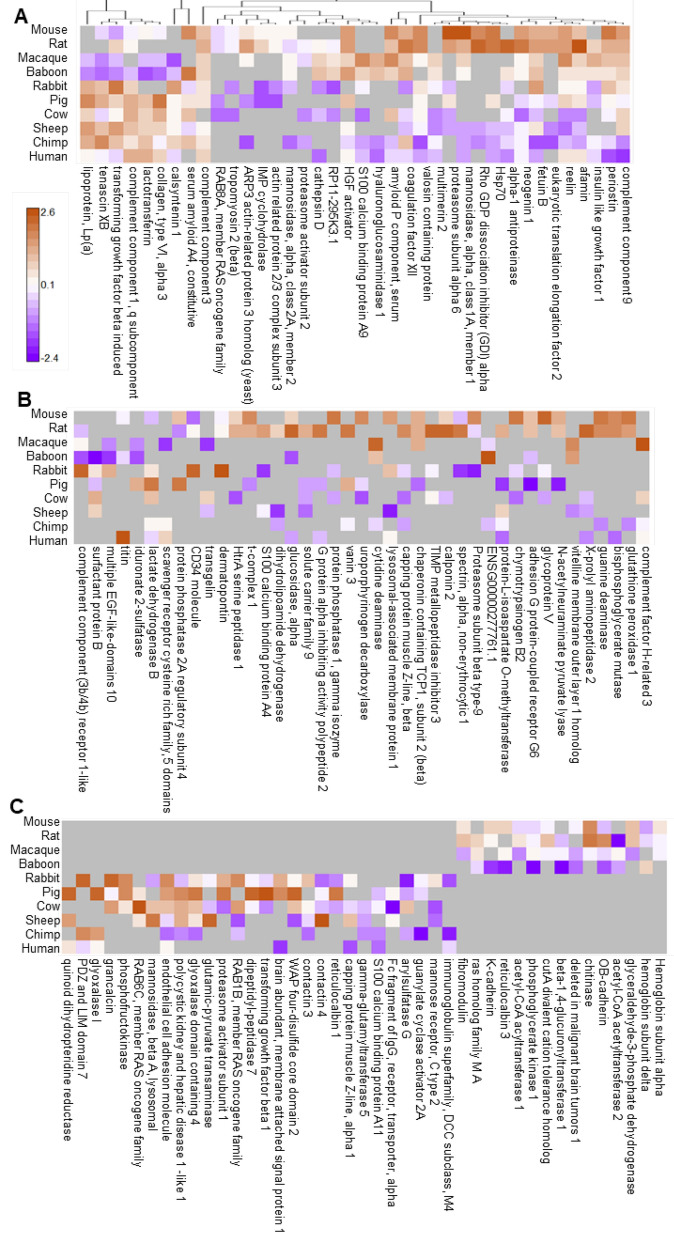
Overview of plasma proteins identified as differentially abundant in comparison of resilient versus sensitive species. Three different comparisons were performed resulting in 122 total proteins identified as differentially abundant. Quantitative protein level values are based upon scaled LFQ intensities combined from peptide level intensities. Color scale represented as scaled quantitative abundance differences with brown representing higher abundance and purple lower abundance for each individual protein. **A** Mapping of 38 proteins at *p* < 0.05, Pearson correlated. **B** Mapping of 41 proteins based upon higher fold-change abundance (± 3.0 in log2 phase, minimum of 4 occurrences), bimodal correlation. **C** Mapping of 43 proteins with yes/no abundance based upon a minimum of 3 species observations, bimodal correlation

Plasma proteins condition cellular responses to LPS (Warren et al. 2010). To assess possible protein differences in the plasmas of sensitive and resilient species, we performed an exploratory proteome analysis of pooled plasma samples for each species in the absence of LPS stimulation, as described in Materials and Methods (Fig. 2A–C, Fig S1). This approach identified a total of 122 proteins that discriminated between the groups by differential abundance and/or detection in one group only (Data S1). These included known modulators of inflammatory cell activity, such as components of the complement and coagulation pathways, TGFβ, and lipoproteins. Since high-density lipoprotein is anti-inflammatory and neutralizes LPS (Tanaka et al. 2020), we purified HDL from each species’ plasma without LPS stimulation, and studied the HDL proteomes using the same analytic approaches. Between 93 and 235 HDL-associated proteins were identified. Eighty-three of these were found in different abundances in the sensitive and resilient groups (Fig S2–3 and Data S2). We also identified lipid components in unstimulated plasma to determine a species-specific lipidomic profile. There were 520 identified and quantified lipids across the species in 22 subclasses (Fig S4), but there was no clear discernable pattern of lipid differentiation between sensitive and resilient species.

### Transcriptomics at baseline shows pre-existing activation in sensitive species

**Fig. 3:**
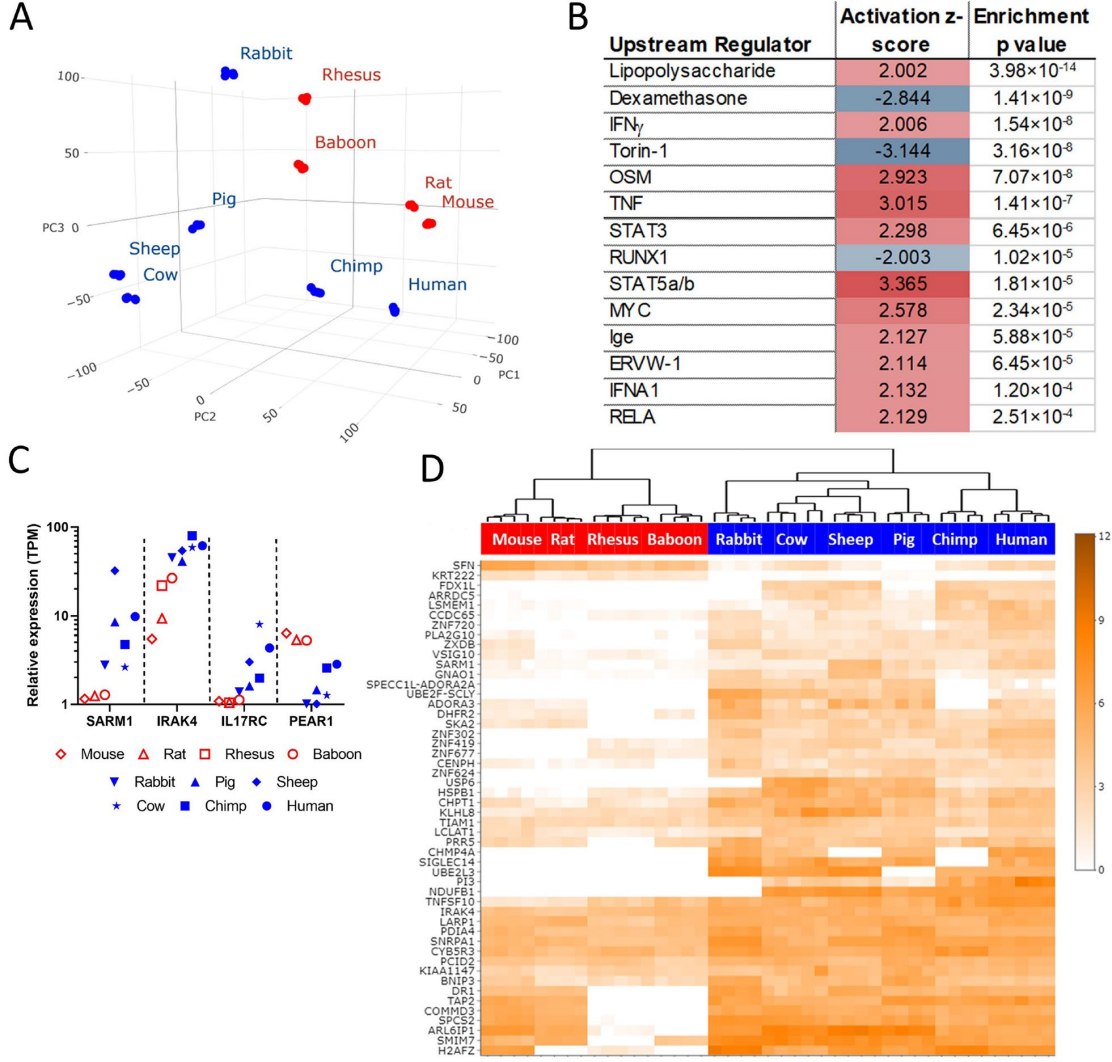
Leukocyte transcriptomes at baseline. **A** Principal component analysis showing overall distribution of individual mRNA abundance between individuals. Resilient animals are indicated with a red dot; blue dots indicate sensitive animals. **B** Regulators of gene expression that are consistent with the differences between expression profiles of resilient and sensitive species. **C** Average expression of four example genes in each species. **D** Clustering of the 50 genes with the greatest expression differences between sensitive and resilient species. Color intensity scale: transcript abundance (log_2_ TPM). Red highlight indicates resilient species, blue highlight indicates sensitive species

We next profiled the gene expression in leukocytes, which are the primary drivers of inflammation at rest and following LPS stimulation. Our earliest time point provides an estimate of the transcriptomic state of the circulating leukocytes in the blood of the different species, i.e. the baseline state, without simulation. At baseline, there was a tendency of resilient species to group together and away from sensitive species across the first three principal components (Fig. 3A), suggesting that different species and their sensitivity to LPS might be distinguished by their resting gene expression.

We sought to identify transcriptomic features that distinguished sensitive and resilient species at baseline. Direct comparison identified 3,012 genes with significantly different expression (twofold difference, FDR < 0.05) between the two groups. Of these, 1,222 were expressed at higher levels and 1790 at lower levels in sensitive species than in resilient species (Data S3). We used Ingenuity Pathway Analysis (Qiagen, Inc) to identify upstream regulators that could mediate this difference (Fig. 3B). Strikingly, the most significantly enriched factor with a positive activation z-score was LPS, suggesting that the gene expression profile of resting leukocytes from sensitive animals resembles that of cells that have already been stimulated with LPS when compared to resting leukocytes from resilient animals. The next highest modifiers with a positive activation Z score were IFNγ, Oncostatin M (OSM), TNF and Stat3, which are all potent mediators of inflammation and immunity. The most enriched modifier with a negative z-score, indicating those agents predicted to reduce genes leading to a sensitive gene profile, was dexamethasone, a potent anti-inflammatory agent. The next most enriched regulators are also linked to inflammation: torin-1 is an inducer of autophagy, which antagonizes inflammation (Deretic and Levine 2018), and RUNX1, which regulates TLR and NF-κB signaling (Bellissimo et al. 2020). These findings suggest that leukocytes in the blood of sensitive animals such as humans have a gene expression pattern consistent with an inflammatory state when compared with leukocytes from resilient animals, even in the absence of stimulation. The proportion of different leukocyte populations varied by species but did not correlate with the sensitivity of the species to LPS (Fig S5), indicating that the differences in gene expression reflect differences in leukocyte biology or cell state, rather than cell number.

More stringent analysis of the data identified 144 genes with completely divergent expression between the classes, i.e. for which there was no overlap in expression between sensitive and resilient animals with identified orthologs (Data S4). Some examples of possible interest are shown in Fig. 3C, which include IRAK4 which is a known central mediator of the cellular response to LPS and other microbial and inflammatory ligands (Suzuki et al. 2002), the MyD88 family member SARM1 (Shanahan et al. 2024; Carty et al. 2019), interleukin 17 receptor (IL17RC) and platelet-derived mediator of clotting and inflammation (PEAR1). The 50 protein coding genes with the largest overall differences between the classes and no overlap in baseline expression between sensitive and resistant animals and are shown in Fig. 3D. Of note, 49 of these genes were expressed more strongly in the sensitive species.

### Response to LPS stimulation reveals differential gene mechanisms in resilient species

**Fig. 4:**
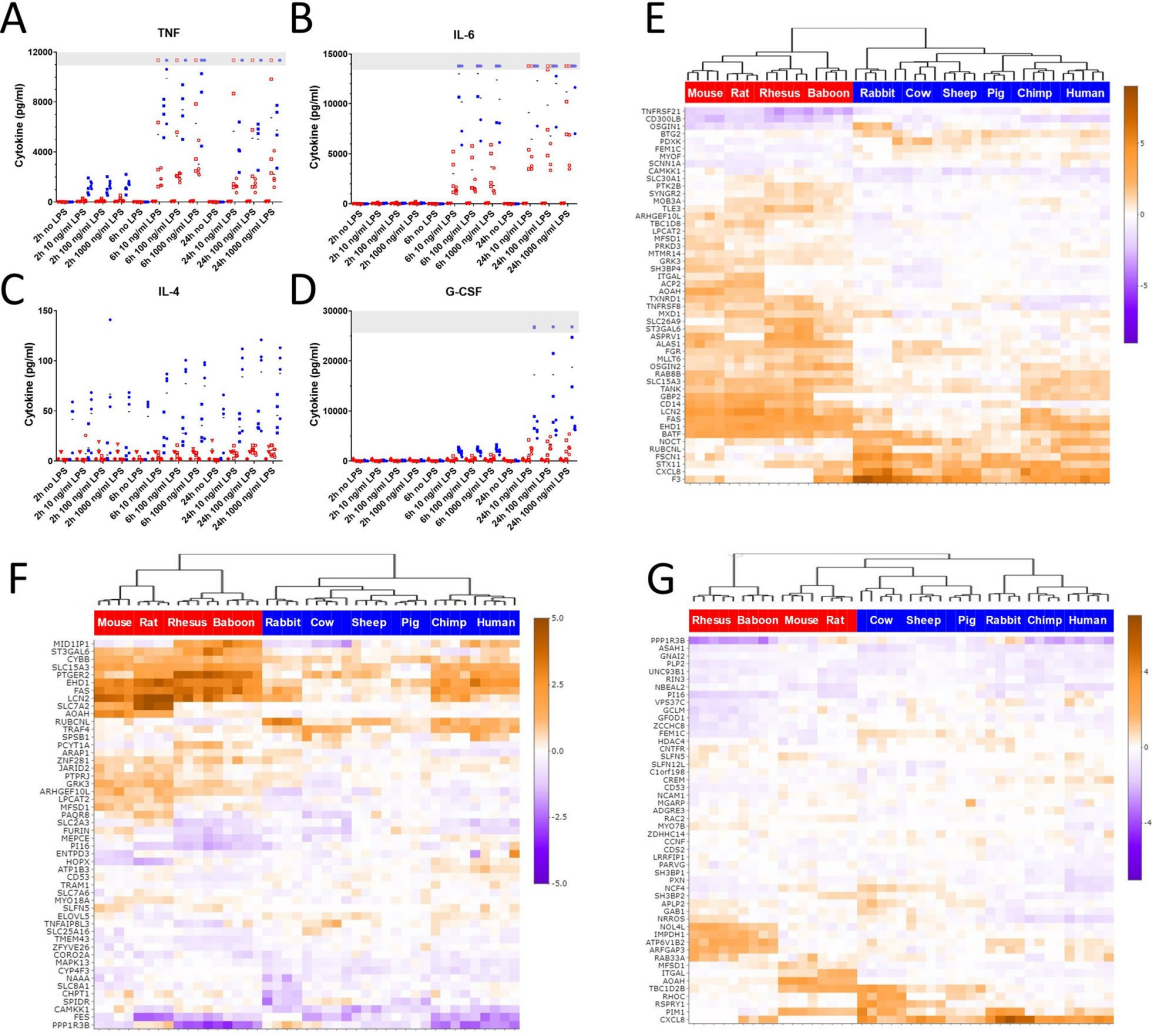
Cytokine and gene expression responses of leukocytes to LPS stimulation. **A–D** Cytokine release in a subset of species, measured by Luminex. Resilient animals are indicated with a red symbol; blue symbols indicate sensitive animals. **E–G** Cluster analysis of the 50 genes with the largest difference in response to 10 ng/mL LPS between sensitive and resilient species after 2 h, 6 h, and 24 h, respectively. Color intensity scale: transcript abundance (log_2_ TPM)

We measured cytokine protein levels in the supernatants of the LPS stimulated whole blood of species for which reagents were available, which included two sensitive species (human and chimpanzee), and four resilient species (baboon, rhesus monkey, mice and rats). Consistent with the in vivo sensitivity profile, the major pro-inflammatory cytokines, IL6 and TNF, as well as G-CSF and IL4, were induced more rapidly and more potently in blood from the sensitive species than the resilient ones (Fig. 4A–D). Similar patterns were found for IFNγ, IL12/23p40, and IL10, but not IL1β (Fig S6). We used targeted proteomics to confirm some of these observations independently of antibody binding (Fig S7).

For gene expression, the effect of LPS treatment on transcription was studied as the fold change for each transcript relative to unstimulated controls (Fig S8A–C). Within each timepoint, individual animals grouped closely with one another, but there was no clear pattern between sensitive and resilient species. The number of differentially expressed genes varied between the 10 species (twofold changes, FDR < 0.05), and more genes increased than decreased in response to LPS for every species (Fig S8D). The greatest response, in terms of numbers of genes regulated, was seen at 6 h for most species. There was a consistent but minimal effect of dose escalation for 10, 100, and 1000 ng/mL LPS, and 10 ng/mL was chosen for further analyses. Notably, the large difference between sensitive and resilient groups in numbers of genes observed at baseline was not reflected in differences between the groups in the number of genes responding to LPS treatment: responses of only 144, 187, and 97 genes were significantly different between resilient and sensitive species at 2, 6, and 24 h respectively (1.5 fold difference, FDR < 0.05) (Data S5-7). Clustering of the top 50 genes for each time point is shown in Fig. 4E–G.

**Fig. 5:**
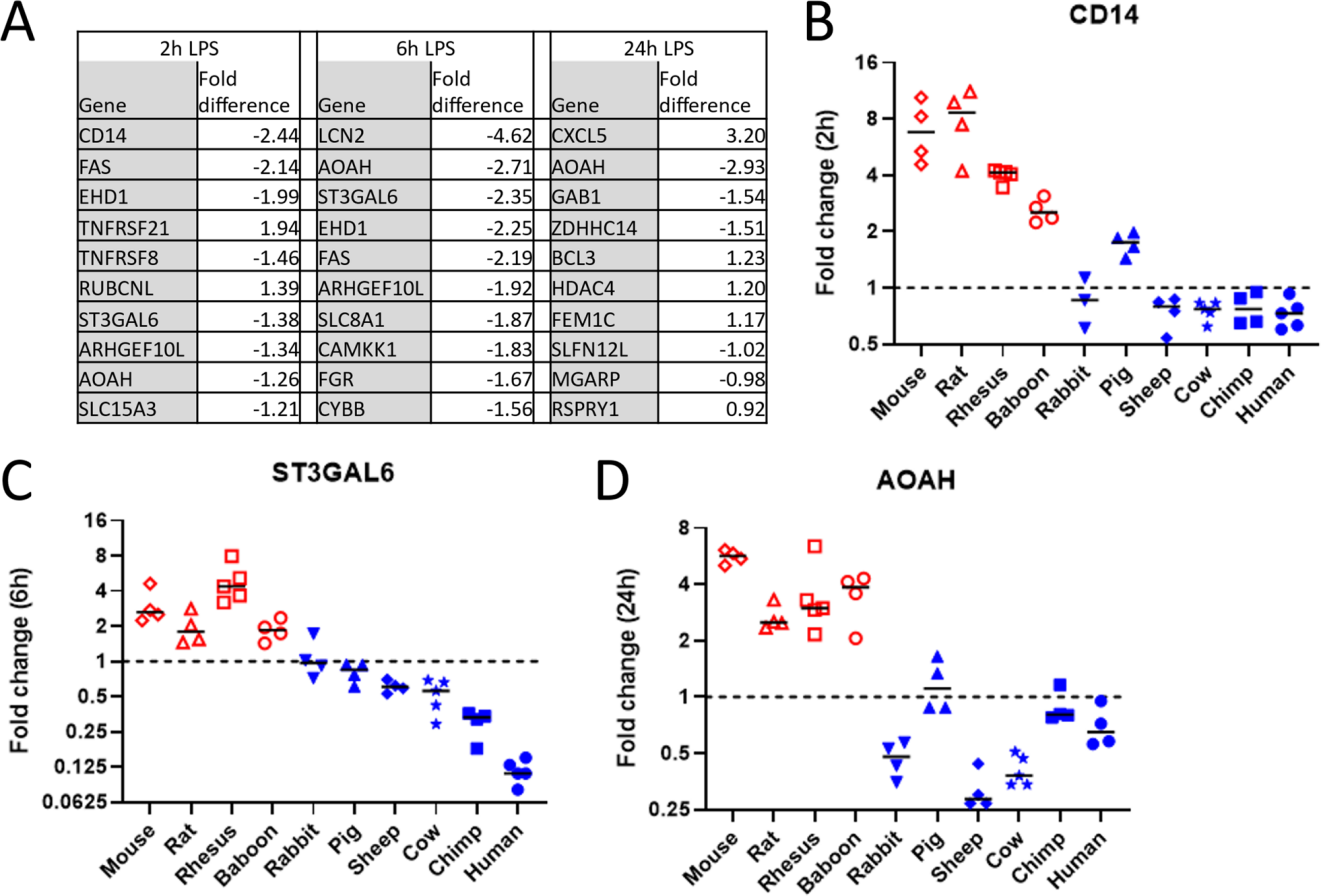
Genes with divergent responses to LPS stimulation. **A** The ten genes at each timepoint with the greatest significant (FDR < 0.05) separation in responses between resilient and sensitive animals. Negative numbers indicate genes that are induced less, or repressed more, by LPS in sensitive species than resilient species. **B–D** Responses of individual species in example genes from each timepoint. Each point depicts the change in expression (fold change in TPM following incubation with and without 10 ng/mL LPS relative to incubation for the same times in media without LPS) in blood from an individual animal. Bars indicate median fold change for each species. Resilient animals are indicated with a red symbol; blue symbols indicate sensitive animals. Dashed line indicates no change on stimulation

We next identified genes where the responses of all individual resilient animals differed from that of all sensitive ones. At 2, 6, and 24 h time points, 48, 60, and 28 genes satisfied this condition, respectively (Data S8-10). The ten genes with maximal differences in LPS-induced fold change between sensitive and resistant species after stimulation are shown in Fig. 5A. Remarkably, at 2 h, 8 of these 10 genes, and at 6 h all 10 genes were induced more strongly in the resilient group, and the protein products of many of these genes are known to play a direct or indirect role in LPS related activities. These include CD14, which is cellular co-receptor for LPS signaling (Zanoni and Granucci 2013), AOAH which is an enzyme that detoxifies LPS through removal of fatty acids (Munford et al. 2020), and ST3GAL6 which is a glycosyltransferase that regulates the stability of another LPS detoxifying enzyme, alkaline phosphorylase (Yang et al. 2018) (Fig. 5 B-D). Five of these were identified at both 2 and 6 h (FAS, EHD1, ST3GAL6, ARHGEF10L, and AOAH) and only one (LCN2) was also significantly different in the baseline analysis. The remaining genes were also notable. Low expression of ARHGEF10L in CD14 + monocytes is associated with increased death from sepsis (Liepelt et al. 2020), and the list included genes related to anti-bacterial activity (LCN2) (Flo et al. 2004), regulation of TLR4 responses (SLC15A3) (Song et al. 2018), LPS sensitivity and autophagy (RUBCNL and SLC8A1) (Zi et al. 2015; Neubert et al. 2020) and apoptosis (FAS, TNFRSF21, TNFRSF8) (Dostert et al. 2019).

## Discussion

LPS-induced inflammation provides a model for inflammation from infectious and non-infectious origins. LPS is also a major driver of inflammation in Gram-negative bacterial infections. We hypothesized that there exists a hierarchy of sensitivity to LPS-induced inflammation in different species and that this difference is reflected in blood plasma or leukocyte gene expression. Our findings suggest that information relating to LPS sensitivity of species is contained in circulating blood leukocytes. We found that: 1. species shown in the literature to be sensitive to LPS have an extensive gene activation pattern in whole blood that is suggestive of LPS stimulation at rest (in the absence of LPS stimulation) when compared to the resilient species, and 2. After LPS stimulation, the gene response that most differs between sensitive and resilient species are a relatively small number of genes that are relevant to LPS detoxification, TLR response, bacterial control, and apoptosis. We speculate that the results reflect different paradigms that have evolved to handle microbial exposure, with sensitive species having constitutively active genes at baseline that are prepared to handle infection at the cost of more potentially damaging inflammation, and resilient species activating certain genes after LPS stimulation that can act to limit the damage associated with LPS and inflammation compared to sensitive species.

It is unclear if the classification of the species that we utilized with respect to their sensitivity to LPS is specific to LPS alone or may also apply to other inflammatory stimuli. This question is important given the large numbers of infectious and non-infectious situations in which inflammation is part of a disease process and because LPS is often used as a stimulant to model inflammation beyond Gram-negative infection. There are no other pro-inflammatory agonists reported in the literature that have been administered in sufficient numbers to permit a similar comparison based upon in vivo sensitivity. In humans, the in vivo transcriptomic response to LPS is similar in many ways to that induced by trauma or burns (Seok et al. 2013). The baseline data most likely reflects genes poised to influence the underlying physiological responses to LPS that are sometimes seen minutes after exposure. Stringent analysis of the genes which differ at baseline between sensitive and resistant species revealed numerous genes involved in inflammation, including IRAK4 which was constitutively expressed in the sensitive species relative to the resistant species and which is an important non-specific central mediator of TLR and IL1 stimulation. We did not find obvious baseline genes that were specific to LPS. While the top hit in the pathway analysis was for LPS, most of the other identified pathways (such as interferon gamma) were non-specific.

In contrast to the baseline results, stringent analysis of genes that were maximally increased or decreased with no overlapping between the two groups two or six hours after LPS stimulation resulted in a list of genes which were mostly increased in the resilient species and which are known to be important in either LPS detoxification or clearance, inflammation, and apoptosis. There was a striking difference in directionality between the genes that had maximum differences between two groups at baseline and after LPS stimulation. Baseline genes that most differed between the two groups were almost exclusively increased in the sensitive group, whereas genes that most differed after LPS stimulation were mostly increased in the resilient group. The factors leading to the physiological responses at different times after LPS stimulation are complex, and it is unclear if these genes or their gene products directly mediate or compensate for pathological inflammation.

Immune cells are conditioned by exposure to the surrounding plasma (Warren et al. 2010), and LPS is rapidly detoxified by binding to HDL (Tanaka et al. 2020), raising the possibility that non-cellular blood components might be related to the species differences in LPS. However, while analysis of the resting plasma proteome, HDL proteome, and lipidome revealed that there were substantial differences between the ten species, we were unable to detect differences between the sensitive and resilient groups that would provide a clear and unifying explanation for the difference in LPS sensitivity in the different species.

There are limitations to our study. The ordering of the sensitivity to the species is based on studies using LPS from different strains of bacteria and different outcome measurements which could have introduced artifacts in the species grouping of responses to LPS. Although minimized as much as possible, there were some differences in the age, sex, prior pathogen exposure and sedation type between the species in these earlier reports as well as in our study. Transcriptomes of all species were related to the human transcriptome. While this was necessary for allowing cross-species comparisons, it can distort the apparent expression of particular genes where there is not 1:1 conservation between species pairs, for example where no ortholog has been identified in a particular species, or where a single human gene is represented by multiple orthologs in another species, or vice versa. The species have differing numbers and proportions of types of leukocytes in their whole blood, so that the studies reflect the net sum of all gene responses in the blood. Although the percentages did not correlate with the species grouping, specific conclusions could be confounded by different cells playing a role in the underlying mechanisms. In addition, our results reflect ex vivo stimulation in the blood compartment only, which does not necessarily reflect results from different tissues. For technical reasons and to ensure that all results between species were strictly comparable, our earliest time point was at two hours. It is possible that stresses to the cells during sample collection, incubation, and erythrocyte lysis could have altered the response even in the absence of LPS. The lack of detection of proinflammatory cytokines such as TNF in samples in which there was no LPS added is reassuring in this regard. Our analyses of pathways were performed using a program that is based upon prior published gene associations to analyze gene pathways. By design the results of these and similar programs include literature biases that populate the medical and research literature. Regardless of these limitations, our data support the hypothesis that species can be characterized by those that respond with high sensitivity and those that are relatively resilient (Fig. 1A) and indicate that information relating to this division is present in blood leukocytes.

To our knowledge, this work represents the first approach to systematically compare the proteomics, lipidomics and transcriptomics between species that are sensitive or resilient to LPS. Humans are at the most sensitive end of the range of species to LPS challenge. The evolutionary advantage for this is not clear, especially considering that many non-human primates are highly resilient, and that their innate immune baseline state does not seem to be associated with any obvious disadvantage. There are however several important implications. First, if innate inflammation is essential for immune defense but at a potential cost of increased inflammatory secondary tissue damage, one might expect humans to be more resilient to infections but also more likely to have inflammatory consequences such as worse symptoms or secondary damage during infection than more resilient species. Second, the results suggest that the choice of a species to mimic any individual inflammatory disease in humans might be improved by tailoring the species to one that mimics humans in relevant disease specific genes. Our data could be used to choose a species in which to model drugs that have a specific gene target (by matching responses with the human response for that gene). Third, it may be possible to utilize these data as a means of discovering new drug targets or as the basis of a new approach to therapeutic modulation of immunity by inducing a state similar to those in resilient species in order to temporarily suppress damaging inflammation during infection or other damaging pro-inflammatory conditions while minimizing immunosuppression.

## Supplementary Information

Additional file 1.

## Data Availability

RNAseq data related to this study has been deposited in GEO, Accession GSE249010. Processed data will be shared on reasonable request to the corresponding authors.

## Abbreviations

IFNγ: Interferon gamma
i.p.: Intraperitoneal
i.v.: Intravenous
LC–MS: Liquid chromatography mass spectrometry
LIQUID: Lipid informed quantitation and identification
LFQ: Protein level quantitative
LPS: Lipopolysaccharide
FDR: False discovery rate
HDL: High density lipoprotein
OSM: Oncostatin
RIN: RNA integrity summary score
SIRS: Systemic inflammatory response syndrome)
SNPRC: Southwest national primate research center
SRM: Selection reaction monitoring
SRV: Simian betaretrovirus
TLE: Total lipid extract
TNF: Tumor necrosis factor
TPM: Transcripts per million
