# Supplementary material for: Molecular profiles of blood from numerous species that differ in sensitivity to acute inflammation
Source: Mol Med. 2024 Dec 28;30:280. doi: 10.1186/s10020-024-01052-x (PMC11681734; doi:10.1186/s10020-024-01052-x)
Supplement: Supplementary file 1 — Additional file 1. [file 10020_2024_1052_MOESM1_ESM.pdf]

Supplementary Figures and Table

Gregory, Han, *et al.*: Multi-omic blood analysis reveals differences in innate inflammatory sensitivity between species.

This supplement contains:

Figs. S1-S8
Table S1
Supplemental references

The following data tables are provided as separate files:

Data S1. Plasma proteins with differing abundance in between sensitive and resilient animals.

Data S2. HDL proteins with differing abundance in between sensitive and resilient animals.

Data S3. Genes with significantly different expression between sensitive and resilient animals in unstimulated leukocytes.

Data S4. Genes with no overlap in expression between sensitive and resilient animals in unstimulated leukocytes.

Data S5. Genes with significantly different expression between sensitive and resilient animals in leukocytes stimulated with 10 ng/mL LPS for 2 hours.

Data S6. Genes with significantly different expression between sensitive and resilient animals in leukocytes stimulated with 10 ng/mL LPS for 6 hours.

Data S7. Genes with significantly different expression between sensitive and resilient animals in leukocytes stimulated with 10 ng/mL LPS for 24 hours.

Data S8. Genes with no overlap in expression between sensitive and resilient animals in leukocytes stimulated with 10 ng/mL LPS for 2 hours.

Data S9. Genes with no overlap in expression between sensitive and resilient animals in leukocytes stimulated with 10 ng/mL LPS for 6 hours.

Data S10. Genes with no overlap in expression between sensitive and resilient animals in leukocytes stimulated with 10 ng/mL LPS for 24 hours.

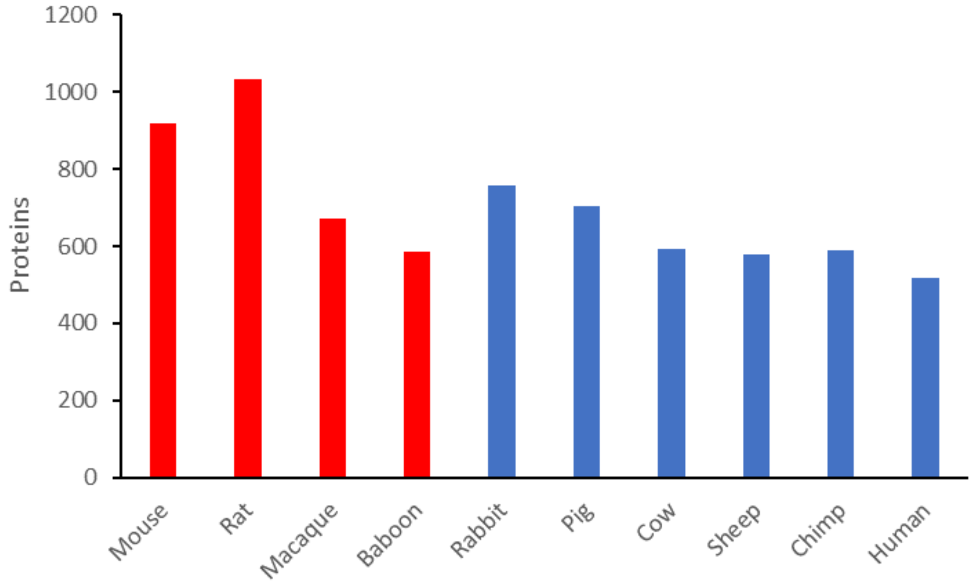

Fig. S1

Total number of proteins identified and quantified in unstimulated whole plasma for each species after LC-MS/MS analysis. Resilient species are indicated in red, sensitive animals in blue.

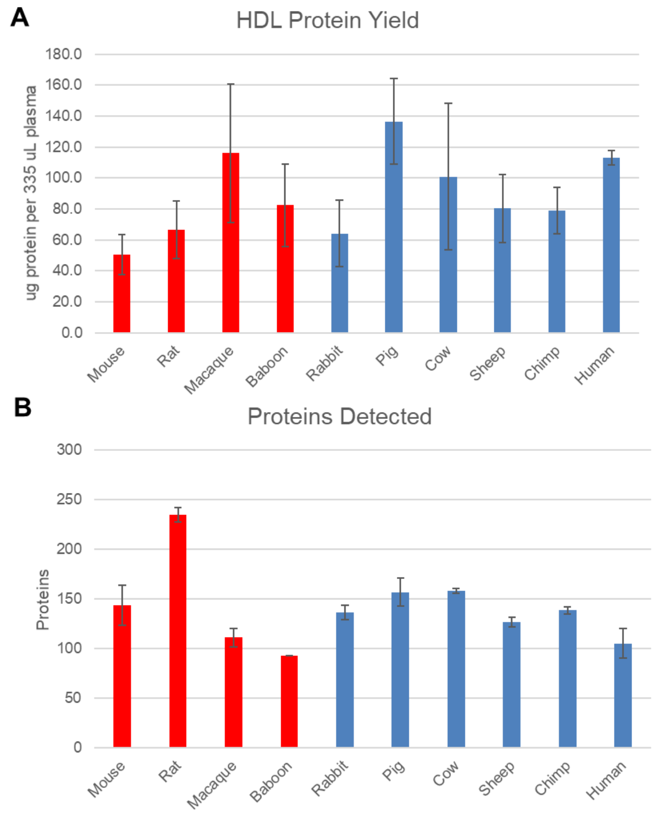

Fig. S2.

Measured protein yields from HDL protein isolation and detection. A) yield of total protein amount per species after isolation of HDL from replicate plasma samples without LPS stimulation. B) unique HDL proteins detected and identified per species after LC-MS/MS analysis. Error bars represent biological triplicate analysis.

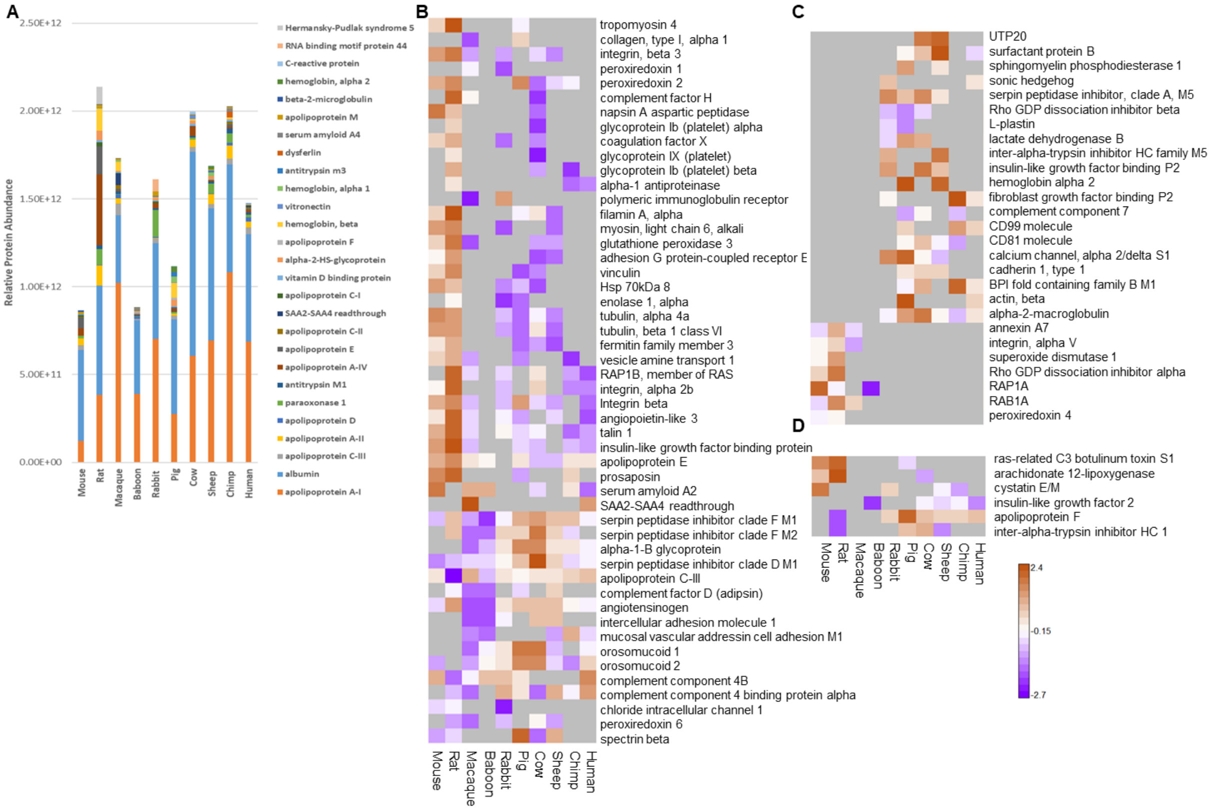

Fig. S3.

Overview of HDL isolated proteins from plasma and identified as differentially abundant in comparison of resilient versus sensitive species. A) top 10 most abundant HDL proteins per species. Three different comparisons were performed (B-D) resulting in 83 total HDL proteins identified as differentially abundant. Quantitative protein level values are based upon scaled LFQ intensities combined from peptide level intensities. Color scale represented as scaled quantitative abundance differences as shown with red representing higher abundance and purple lower abundance for each individual protein. B) mapping of 50 proteins at *p* <0.05, Pearson correlated. C) mapping of 27 proteins with yes/no abundance based upon a minimum of 3 species observations, bimodal correlation. D) mapping of 6 proteins based upon higher fold-change abundance (+/-3.0 in $\log_2$ phase, minimum of 4 occurrences), bimodal correlation.

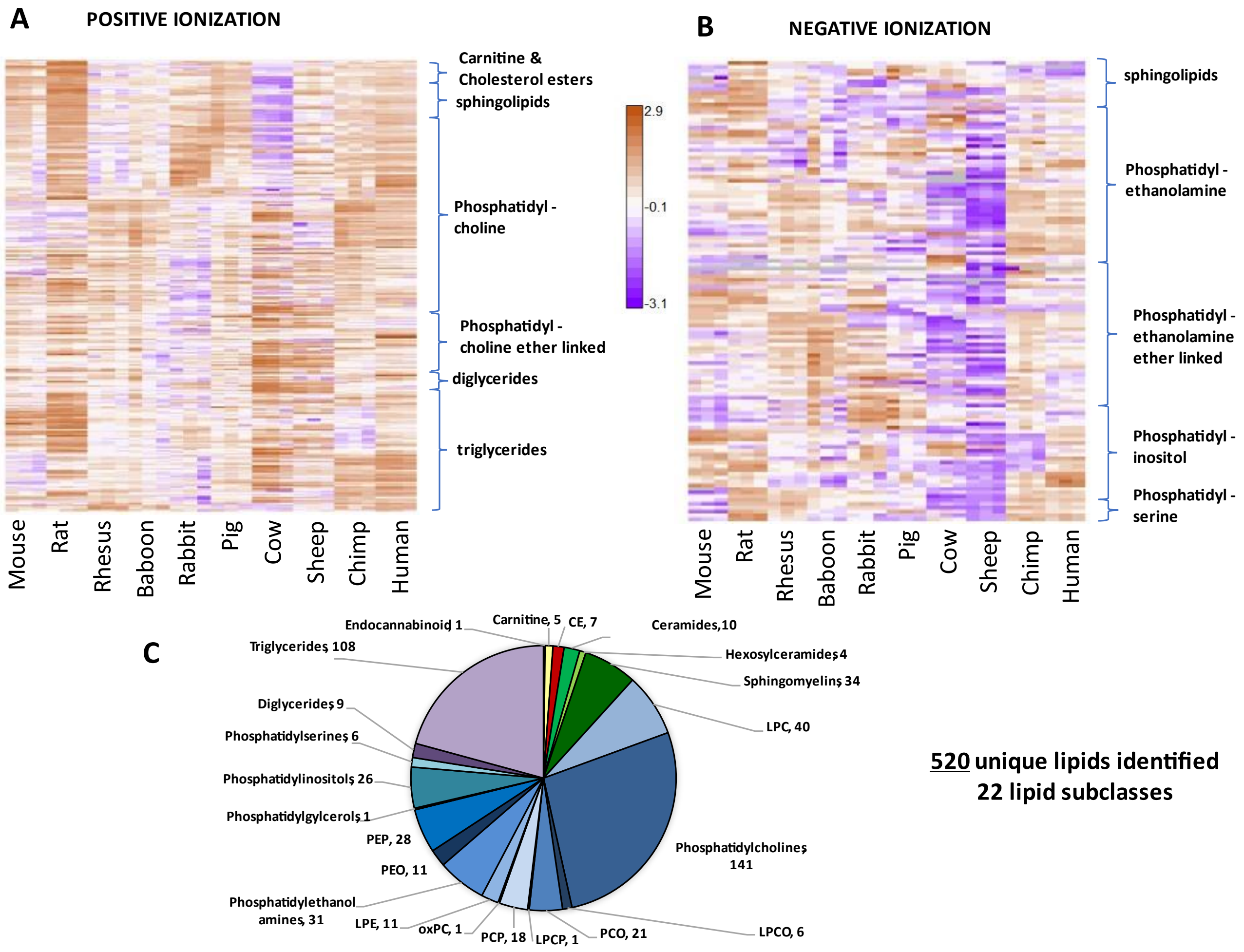

Fig. S4.

Plasma lipidomes. Abundance of individual lipids that form positive (A) or negative (B) ions detected and quantified via LC-ESI-MS/MS is shown for each of 3 individuals per species, relative to the global mean signal intensity for each lipid. (C) Total number of distinct lipids identified, by lipid subclass. CE: cholesterol esters; LPC: lyso-phosphatidylcholines; LPCO: monoalkylglycerophosphocholines; PCO 1-alkyl,2-acylglycerophosphocholines; LPCP: 1Z-alkenylglycerophosphocholines; PCP: 1-(1Z-alkenyl),2-acylglycerophosphocholines; oxPC: oxidized phosphatidylcholines; LPE: lyso-phosphatidylethanolamines.

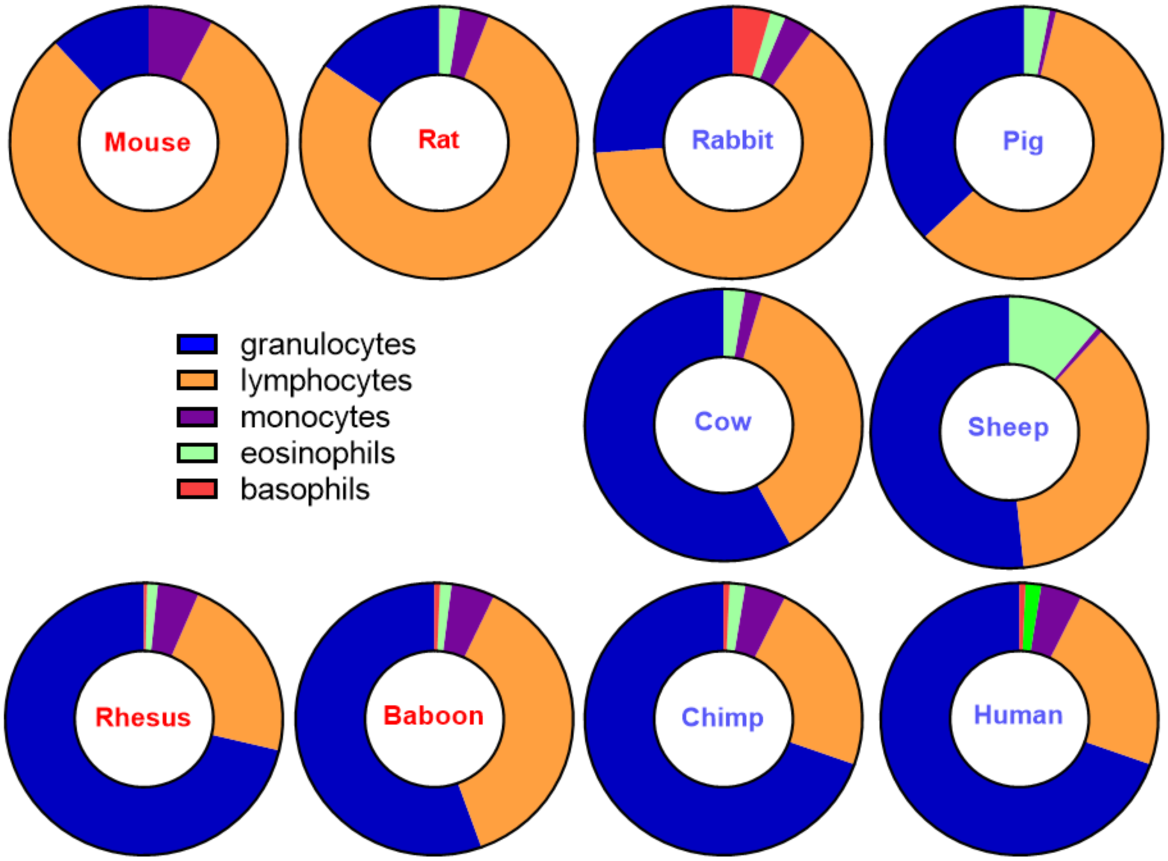

Fig. S5.

Blood differential counts obtained from the blood used in this study. Due to safety limitations, data was not obtained from the human subjects; established clinical reference values are given instead.

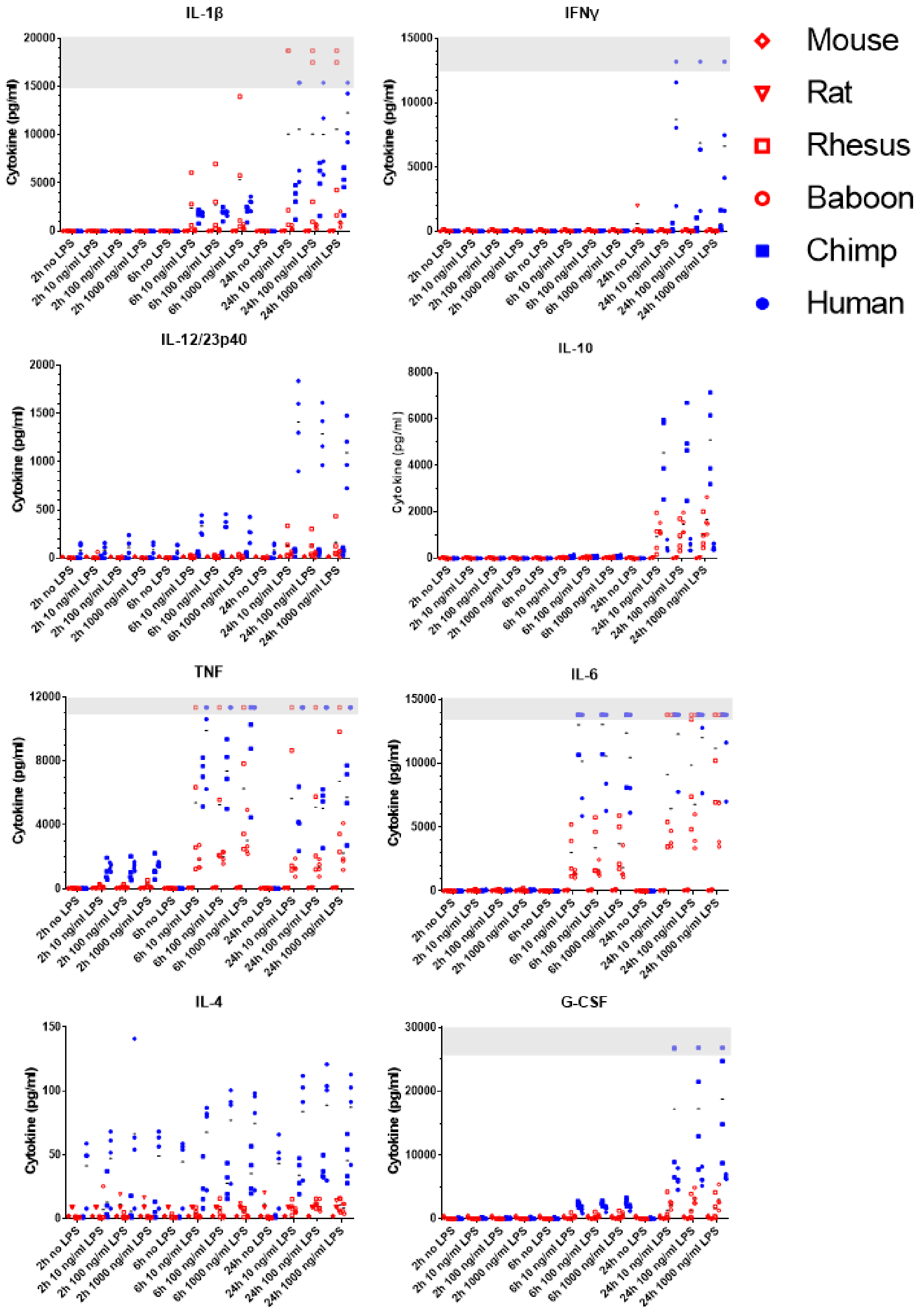

Fig. S6.

Cytokine release following LPS stimulation in the subset of species where antibodies were available, measured by Luminex. Resilient animals are indicated with a red symbol; blue symbols indicate sensitive animals. Black bars indicate mean values for each species. Grey bars indicate readings above quantifiable range.

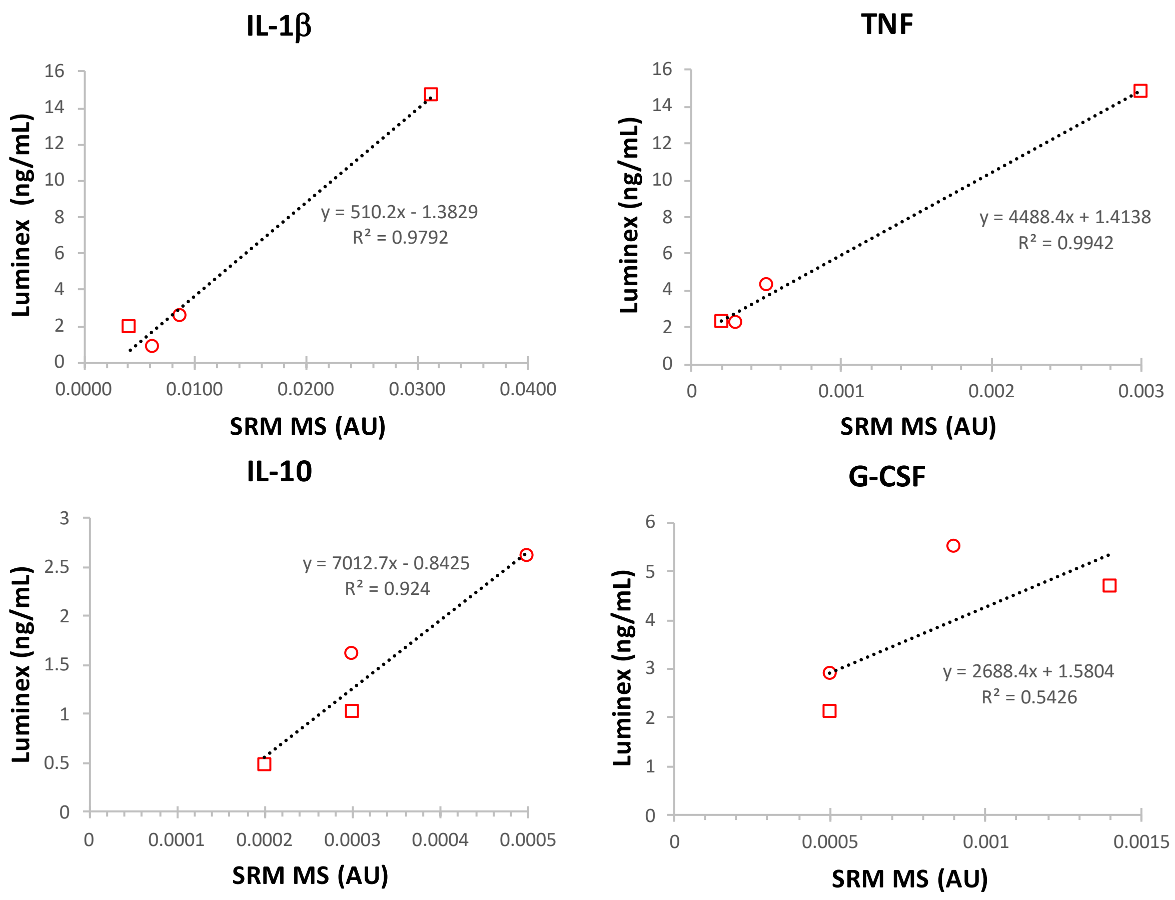

Fig. S7.

Validation of cytokine measurements. Concentrations of the indicated cytokines were established by sequence specific targeted SRM MS of unique peptides for two rhesus (squares) and two baboon (circles) plasma samples. Concentrations are expressed relative to peptide standard in arbitrary units (AU). Values are correlated with those obtained by Luminex for the same samples.

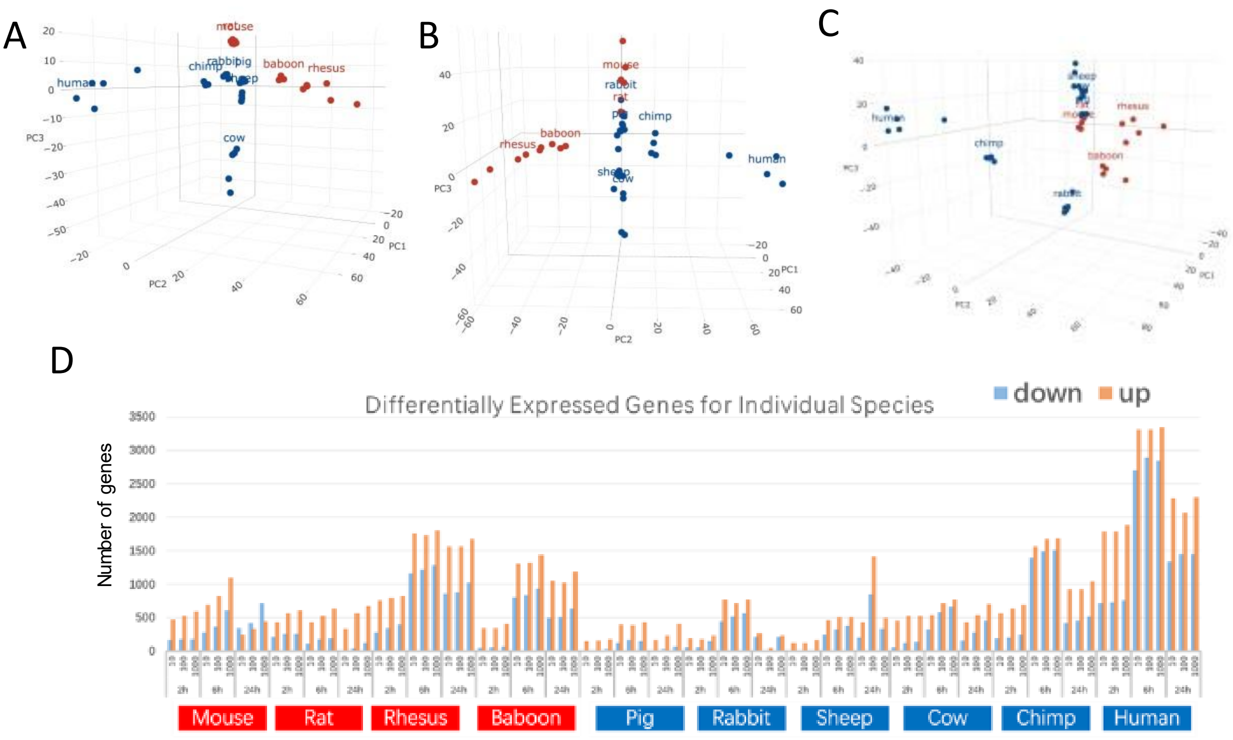

Fig. S8.

Gene expression responses of leukocytes to LPS stimulation. Principal component analysis showing overall distribution of fold change in individual mRNA abundance at A) 2h, B) 6h, or C) 24h following 10 ng/mL LPS stimulation. D) Number of genes significantly (2-fold change, FDR<0.05) changed relative to baseline at each time point after stimulation with 10, 100, or 1000 ng/mL LPS.

Table S1.
Summary of animals bled in this study

Species	Age	Sex	Samples	Sedation	Source	Housing
Mouse	10 week	Male	4× pools of 25	Ketamine-xylazine	Cardiac	Specific pathogen-free barrier facility
Rat	14 week	Male	4× pools of 4	Isoflurane	Cardiac	Specific pathogen-free barrier facility
Rhesus	4 years	Male	5× individuals	Ketamine	Venous	Open air, specific pathogen-free
Baboon	13-18 years	Male	4× individuals	Ketamine	Venous	Open air, specific pathogen-free
Pig	Adult	Male (castrated)	4× individuals	None	Venous	Farm
Rabbit	22 week	Male	4× individuals	Isoflurane	Cardiac	Specific pathogen-free barrier facility
Sheep	Adult	Female	4× individuals	None	Venous	Farm
Cow	Adult	Female	5× individuals	None	Venous	Farm
Chimp	19-26 years	Male	4× individuals	Telazol/ xylazine	Venous	Open air, specific pathogen-free
Human	Adult	Male	5× individuals	None	Venous	Unrestricted

Table S2.
Details of studies used to define species as resilient or sensitive to 1 mg/kg LPS.

Species	Strain	Route	Study	Reference
Mouse	NMRI	i.p.	McCuskey 1984	(1)
Mouse	Balb/c	i.v.	Remick 1995	(2)
Mouse	CD-1	i.v.	Craig 1974	(3)
Mouse	C57/B6	i.p.	Hill 1992	(4)
Mouse	Swiss Webster	i.p.	Su 1997	(5)
Rat		i.p.	Berczi 1966	(6)
Rat	PVG	i.p,	Clark 1982	(7)
Rat	Wistar	i.v	McCuskey 1984	(1)
Rat	Sprague-Dawley	i.v.	Whalley 1992	(8)
Rat	Wistar	i.v.	Kustanova 2006	(9)
Rhesus		i.v.	Sheagren 1967	(10)
Rhesus		i.v.	Coalson 1970	(11)
Macaque		i.v.	Dinbar 1971	(12)
Rhesus		i.v.	Fiser 1974	(13)
Rhesus		i.v.	Premaratne 1995	(14)
Baboon		i.v.	Fletcher 1980	(15)
Baboon		i.v.	Casey 1985	(16)

Baboon		i.v.	Fischer 1992	(17)
Baboon		i.v.	Kneidinger 1996	(18)
Rabbit		i.p.	Berczi 1966	(6)
Rabbit	NZ White	i.v.	Mathison 1988	(19)
Rabbit	NZ White	i.v.	Barrett 1988	(20)
Rabbit	NZ White	i.v.	Whalley, 1992	(8)
Rabbit	NZ White	i.v.	Semeraro 1993	(21)
Rabbit	NZ White	i.v.	Carvalho 1997	(22)
Rabbit	NZ White	i.v.	Kishnamurti	(23)
Pig	Yucatan minipig	i.v.	Hand 1983	(24)
Pig	Minipig	i.v., cumulative	Beller 1985	(25)
Pig	Yorkshire	i.v.	Goldfarb, 1986	(26)
Pig	Mixed breed	i.v.	Schrauwen, 1988	(27)
Pig		Intra-arterial, cumulative	Mozes, 1991	(28)
Pig	Cross-bred	i.v.	Majetschak, 2004	(29)
Cow		i.p.	Berczi, 1966	(6)
Cow	Holstein	i.v.	Ohtsuka, 1997	(30)
Cow	Holstein	i.v.	Gerros, 1995	(31)
Cow	Holstein	i.v.	Yilmaz, 2016	(32)
Sheep		i.v.	Esbenshade, 1982	(33)
Sheep	Mixed breed	Intra-arterial	Golenbock,1987	(34)
Sheep	Suffolk/Merino	i.v, cumulative	Doty, 1988	(35)
Sheep	Suffolk	i.v.	Whyte, 1989	(36)
Sheep	Mixed breed	i.v., cumulatuve	Perkowski, 1996	(37)
Sheep	Mixed breed	i.v., cumulative	Schiffer, 2002	(38)
Chimp		i.v.	Tully, 1965	(39)
Chimp		i.v.	Van der Poll, 2008	(40)
Chimp		i.v.	Van fer Poll, 2008	(41)
Human		i.v.	Sauter, 1980	(42)
Human		i.v.	Elin, 1981	(43)
Human		i.v.	Van Deventer, 1990	(44)
Human		i.v.	Martich, 1991	(45)
Human		i.v.	Taveira da Silva, 1993	(46)

Supplemental references

1. McCuskey,R.S., McCuskey,P.A., Urbaschek,R. and Urbaschek,B. (1984) Species differences in Kupffer cells and endotoxin sensitivity. *Infection and immunity*, **45**, 278–80.

2. Remick,D., Manohar,P., Bolgos,G., Rodriguez,J., Moldawer,L. and Wollenberg,G. (1995) Blockade of tumor necrosis factor reduces lipopolysaccharide lethality, but not the lethality of cecal ligation and puncture. *Shock (Augusta, Ga.)*, **4**, 89–95.

3. Craig,W.A., Turner,J.H. and Kunin,C.M. (1974) Prevention of the generalized Shwartzman reaction and endotoxin lethality by polymyxin B localized in tissues. *Infection and immunity*, **10**, 287–92.

4. Hill,M.R. and McCallum,R.E. (1992) Identification of tumor necrosis factor as a transcriptional regulator of the phosphoenolpyruvate carboxykinase gene following endotoxin treatment of mice. *Infection and immunity*, **60**, 4040–50.

5. Su,D., Roth,R.I., Yoshida,M. and Levin,J. (1997) Hemoglobin increases mortality from bacterial endotoxin. *Infection and immunity*, **65**, 1258–66.

6. Berczi,I., Bertók,L. and Bereznai,T. (1966) Comparative studies on the toxicity of *Escherichia coli* lipopolysaccharide endotoxin in various animal species. *Canadian Journal of Microbiology*, **12**, 1070–1071.

7. Clark,I.A. (1982) Correlation between susceptibility to malaria and babesia parasites and to endotoxicity. *Transactions of the Royal Society of Tropical Medicine and Hygiene*, **76**, 4–7.

8. Whalley,E.T., Solomon,J.A., Modafferi,D.M., Bonham,K.A. and Cheronis,J.C. (1992) CP-0127, a novel potent bradykinin antagonist, increases survival in rat and rabbit models of endotoxin shock. *Agents and actions. Supplements*, **38 (Pt 3)**, 413–20.

9. Kustanova,G.A., Murashev,A.N., Karpov,V.L., Margulis,B.A., Guzhova,I. V, Prokhorenko,I.R., Grachev,S. V and Evgen’ev,M.B. (2006) Exogenous heat shock protein 70 mediates sepsis manifestations and decreases the mortality rate in rats. *Cell stress & chaperones*, **11**, 276–86.

10. Sheagren,J.N., Wolff,S.M. and Shulman,N.R. (1967) Febrile and hematologic responses of rhesus monkeys to bacterial endotoxin. *The American journal of physiology*, **212**, 884–90.

11. Coalson,J.J., Hinshaw,L.B. and Guenter,C.A. (1970) The pulmonary ultrastructure in septic shock. *Experimental and Molecular Pathology*, **12**, 84–103.

12. Dinbar,A., Rapaport,S.I., Patch,M.J., Grant,W. and Fonkalsrud,E.W. (1971) Hematologic effects of endotoxin on the macaque monkey. *Surgery*, **70**, 596–603.

13. Fiser,R.H., Denniston,J.G. and Beisel,W.R. (1974) Endotoxemia in the Rhesus Monkey: Alterations in Host Lipid and Carbohydrate Metabolism. *Pediatric Research*, **8**, 13–17.

14. Premaratne,S., May,M.L., Nakasone,C.K. and McNamara,J.J. (1995) Pharmacokinetics of endotoxin in a rhesus macaque septic shock model. *The Journal of surgical research*, **59**, 428–32.

15. Fletcher,J.R. and Ramwell,P.W. (1980) Indomethacin treatment following baboon endotoxin shock improves survival. *Advances in shock research*, **4**, 103–11.

16. Casey,L.C., Fletcher,J.R., Zmudka,M.I. and Ramwell,P.W. (1985) The role of thromboxane in primate endotoxin shock. *Journal of Surgical Research*, **39**, 140–149.

17. Fischer,E., Marano,M.A., Van Zee,K.J., Rock,C.S., Hawes,A.S., Thompson,W.A., DeForge,L., Kenney,J.S., Remick,D.G. and Bloedow,D.C. (1992) Interleukin-1 receptor blockade improves survival and hemodynamic performance in Escherichia coli septic shock, but fails to alter host responses to sublethal endotoxemia. *The Journal of Clinical Investigation*, **89**, 1551–1557.

18. Kneidinger,R., Bahrami,S., Redl,H., Schlag,G. and Robinson,M. (1996) Comparison of endothelial activation during endotoxic and posttraumatic conditions by serum analysis of soluble e-selectin in nonhuman primates. *Journal of Laboratory and Clinical Medicine*, **128**, 515–519.

19. Mathison,J.C., Wolfson,E. and Ulevitch,R.J. (1988) Participation of tumor necrosis factor in the mediation of gram negative bacterial lipopolysaccharide-induced injury in rabbits. *The Journal of clinical investigation*, **81**, 1925–37.

20. Barrett,T.J., Potter,M.E. and Wachsmuth,I.K. (1989) Bacterial endotoxin both enhances and inhibits the toxicity of Shiga-like toxin II in rabbits and mice. *Infection and immunity*, **57**, 3434–7.

21. Semeraro,N., Triggiani,R., Montemurro,P., Cavallo,L.G. and Colucci,M. (1993) Enhanced endothelial tissue factor but normal thrombomodulin in endotoxin-treated rabbits. *Thrombosis Research*, **71**, 479–486.

22. Carvalho,G.L., Wakabayashi,G., Shimazu,M., Karahashi,T., Yoshida,M., Yamamoto,S., Matsushima,K., Mukaida,N., Clark,B.D., Takabayashi,T., *et al.* (1997) Anti-interleukin-8 monoclonal antibody reduces free radical production and improves hemodynamics and survival rate in endotoxic shock in rabbits. *Surgery*, **122**, 60–68.

23. Krishnamurti,C., Carter,A.J., Maglasang,P., Hess,J.R., Cutting,M.A. and Alving,B.M. (1997) Cardiovascular toxicity of human cross-linked hemoglobin in a rabbit endotoxemia model. *Critical care medicine*, **25**, 1874–80.

24. Hand,M.S., Fettman,M.J., Chandrasena,L.G., Cleek,J.L. and Phillips,R.W. (1983) Endotoxin dose. I. Hemodynamic, metabolic, and lethal consequences in Yucatan minipigs. *The American journal of physiology*, **244**, E385-98.

25. Beller,F.K., Schmidt,E.H., Holzgreve,W. and Hauss,J. (1985) Septicemia during pregnancy: A study in different species of experimental animals. *American Journal of Obstetrics and Gynecology*, **151**, 967–975.

26. Goldfarb,R.D., Nightingale,L.M., Kish,P., Weber,P.B. and Loegering,D.J. (1986) Left ventricular function during lethal and sublethal endotoxemia in swine. *The American journal of physiology*, **251**, H364-73.

27. Schrauwen,E., Cox,E. and Houvenaghel,A. (1988) Escherichia coli sepsis and endotoxemia in conscious young pigs. *Veterinary research communications*, **12**, 295–303.

28. Mózes,T., Ben-Efraim,S., Tak,CornéJ.A.M., Heiligers,J.P.C., Saxena,P.R. and Bonta,I.L. (1991) Serum levels of tumor necrosis factor determine the fatal or non-fatal course of endotoxic shock. *Immunology Letters*, **27**, 157–162.

29. Majetschak,M., Cohn,S.M., Nelson,J.A., Burton,E.H., Obertacke,U. and Proctor,K.G. (2004) Effects of exogenous ubiquitin in lethal endotoxemia. *Surgery*, **135**, 536–43.

30. OHTSUKA,H., HIGUCHI,T., MATSUZAWA,H., SATO,H., TAKAHASHI,K., TAKAHASHI,J. and YOSHINO,T. (1997) Inhibitory Effect of LPS-Induced Tumor Necrosis Factor in Calves Treated with Chlorpromazine or Pentoxifylline. *Journal of Veterinary Medical Science*, **59**, 1075–1077.

31. Gerros,T.C., Semrad,S.D. and Proctor,R.A. (1995) Alterations in clinical, hematological and metabolic variables in bovine neonatal endotoxemia. *Canadian journal of veterinary research = Revue canadienne de recherche veterinaire*, **59**, 34–9.

32. Yilmaz,Z., Eralp Inan,O., Kocaturk,M., Baykal,A.T., Hacariz,O., Hatipoglu,I., Tvarijonaviciute,A., Cansev,M., Ceron,J. and Ulus,I.H. (2016) Changes in serum proteins after endotoxin administration in healthy and choline-treated calves. *BMC Veterinary Research*, **12**, 210.

33. Esbenshade,A.M., Newman,J.H., Lams,P.M., Jolles,H. and Brigham,K.L. (1982) Respiratory failure after endotoxin infusion in sheep: lung mechanics and lung fluid balance. *Journal of Applied Physiology*, **53**.

34. Golenbock,D.T., Will,J.A., Raetz,C.R. and Proctor,R.A. (1987) Lipid X ameliorates pulmonary hypertension and protects sheep from death due to endotoxin. *Infection and immunity*, **55**, 2471–6.

35. Doty,S., Traber,L., Herndon,D., Kimura,R., Lubbesmeyer,H., Davenport,S. and Traber,D. (1988) Beta endorphin, a vasoconstrictor during septic shock. *The Journal of trauma*, **28**, 131–9.

36. Whyte,R.I., Warren,H.S., Greene,E., Glennon,M.L., Robinson,D.R. and Zapol,W.M. (1989) Tolerance to low-dose endotoxin in awake sheep. *Journal of Applied Physiology*, **66**.

37. Perkowski,S.Z., Sloane,P.J., Spath,J.A., Elsasser,T.H., Fisher,J.K. and Gee,M.H. (1996) TNF-alpha and the pathophysiology of endotoxin-induced acute respiratory failure in sheep. *Journal of applied physiology (Bethesda, Md. : 1985)*, **80**, 564–73.

38. Schiffer,E.R.C., Reber,G., De Moerloose,P. and Morel,D.R. (2002) Evaluation of unfractionated heparin and recombinant hirudin on survival in a sustained ovine endotoxin shock model. *Critical care medicine*, **30**, 2689–99.

39. Tully,J.G., Gaines,S. and Tigertt,W.D. (1965) Studies on Infection and Immunity in Experimental Typhoid Fever VI. Response of Chimpanzees to Endotoxin and the Effect of Tolerance on Resistance to Oral Challenge. *The Journal of Infectious Diseases*, **115**, 445–455.

40. van der Poll,T., Jansen,J., van Leenen,D., von der Möhlen,M., Levi,M., ten Cate,H., Gallati,H., ten Cate,J.W. and van Deventer,S.J. (1993) Release of soluble receptors for tumor necrosis factor in clinical sepsis and experimental endotoxemia. *The Journal of infectious diseases*, **168**, 955–60.

41. VAN DER POLL,T., LEVI,M., CATE,H. TEN, JANSEN,J., BIEMOND,B.J., HAAGMANS,B.L., EERENBERG,A., VAN DEVENTER,S.J.H., HACK,C.E. and CATE,J.W. TEN (2008) Effect of postponed treatment with an anti-tumour necrosis factor (TNF) F(ab')2 fragment on endotoxin-induced cytokine and neutrophil responses in chimpanzees. *Clinical and Experimental Immunology*, **100**, 21–25.

42. Sauter,C. and Wolfensberger,C. (1980) Interferon in human serum after injection of endotoxin. *The Lancet*, **316**, 852–853.

43. Elin,R.J., Wolff,S.M., McAdam,K.P.W.J., Chedid,L., Audibert,F., Bernard,C. and Oberling,F. (1981) Properties of Reference Escherichia coli Endotoxin and Its Phthalylated Derivative in Humans. *Journal of Infectious Diseases*, **144**, 329–336.

44. van Deventer,S.J., Büller,H.R., ten Cate,J.W., Aarden,L.A., Hack,C.E. and Sturk,A. (1990) Experimental endotoxemia in humans: analysis of cytokine release and coagulation, fibrinolytic, and complement pathways. *Blood*, **76**, 2520–6.

45. Martich,G.D., Danner,R.L., Ceska,M. and Suffredini,A.F. (1991) Detection of interleukin 8 and tumor necrosis factor in normal humans after intravenous endotoxin: the effect of antiinflammatory agents. *Journal of Experimental Medicine*, **173**.

46. Taveira da Silva,A.M., Kaulbach,H.C., Chuidian,F.S., Lambert,D.R., Suffredini,A.F. and Danner,R.L. (1993) Shock and Multiple-Organ Dysfunction after Self-Administration of Salmonella Endotoxin. *New England Journal of Medicine*, **328**, 1457–1460.
